# Rethinking the path from evidence to decision-making

**DOI:** 10.1186/s13584-023-00559-8

**Published:** 2023-03-27

**Authors:** Alon Rasooly, Eliana Ben-Sheleg, Nadav Davidovitch, Moriah Ellen

**Affiliations:** Department of Health Policy and Management, Guilford Glazer Faculty of Business and Management and School of Public Health, Faculty of Health Sciences, Ben-Gurion University of the Negev, Beer-Sheva, Israel

**Keywords:** Evidence-informed policy, Health policy, Implementation science, International workshop, Knowledge transfer and exchange

## Abstract

Evidence-informed decision-making is increasingly recognized as a standard for policymaking in many fields, including public health. However, many challenges exist in identifying the appropriate evidence, disseminating it to different stakeholders, and implementing it in various settings. The Israel Implementation Science and Policy Engagement Centre (IS-PEC) was established at Ben-Gurion University of the Negev to “bridge the gap” between scientific research and policy. As an illustrative case study, IS-PEC is conducting a scoping review on strategies to engage senior citizens in Israel when developing health policy. In May 2022, IS-PEC brought together international experts and Israeli stakeholders to increase knowledge in the field of evidence-informed policy, develop a research agenda, strengthen international collaborations, and create a community for sharing experience, research, and best practices. Panelists presented the importance of communicating clear, accurate bottom-line messages with the media. Also, they highlighted the once-in-a-generation opportunity to promote the uptake of evidence in public health due to the increased public interest in evidence-informed policymaking post-COVID-19 pandemic and the need to build systems and centers to support the systematic use of evidence. Group discussions focused on various aspects of communication, including challenges and strategies when communicating to policymakers, understanding the nuances of communication between scientists, journalists, and the public, and some ethical issues surrounding data visualization and infographics. Panelists participated in a passionate debate regarding whether and how values play a role when conducting, analyzing, and communicating evidence. Takeaway lessons from the workshop included that going forward, Israel must create lasting systems and a sustainable environment for evidence-informed policy. Novel and interdisciplinary academic programs must be developed to train future policymakers in various fields, including public health, public policy, ethics, communication, social marketing, and infographics. Sustainable professional relationships between journalists, scientists, and policymakers must be fostered and strengthened based on mutual respect and a shared commitment to creating, synthesizing, implementing, and communicating high-quality evidence to serve the public and individual wellbeing.

**Supplementary Information:**

The online version contains supplementary material available at 10.1186/s13584-023-00559-8.

## The evidence-informed policy workshop: rethinking the path from evidence to decision making

Evidence-informed decision-making and policymaking are increasingly becoming recognized globally as a standard in developing programs and policy interventions. However, many challenges exist in identifying the appropriate evidence, disseminating it to different stakeholders, and implementing it in and collaborating across different settings [4, 9]. The void between what science knows and what practice does has become known as the “know-do gap” [12]. This results in the failure of organizations and players to successfully implement well-established solutions or policies which could solve or ameliorate the problems at hand. The phenomenon exists across multiple fields, including but not limited to healthcare, education, and environmental policy.

The Israel Implementation Science and Policy Engagement Centre (IS-PEC) was established at Ben-Gurion University of the Negev in the summer of 2021 to “bridge the gap” between science and policy. IS-PEC is the first of its kind in Israel and one of the few centres worldwide focusing on implementation science and policy engagement [5].

This research centre was founded by Moriah Ellen, a professor of health systems and policy at Ben-Gurion University, and is led by an interdisciplinary steering committee and internationally renowned strategic advisors. By bringing together the fields of Implementation Science (IS) and Knowledge Transfer and Exchange (KTE), the centre seeks to prepare future leaders in the most advanced methods. The centre also collaboratively works to build a strong culture of evidence-based implementation and evidence-informed policy in Israel while contributing to global knowledge.

IS-PEC conducts impactful national and international research and investigates stakeholder and citizen engagement in policymaking, monitoring and evaluating existing KTE practices, and evaluating healthcare professionals’ perceptions of issues on IS and KTE. As an illustrative case study, in Israel, IS-PEC is conducting a scoping review to identify what is known regarding strategies to engage senior citizens and informal caregivers when developing health policy. Through mapping available strategies, IS-PEC uses updated methodological guidance to provide an evidence-based foundation to guide senior citizens’ engagement in health policy development [6]. Internationally, IS-PEC’s researchers recently led a knowledge translation initiative with collaborators from Shanghai, China, in implementing evidence-informed quality indicators for primary diabetes care [13].

In May 2022, IS-PEC held a two-day workshop funded by the Bat-Sheva de Rothschild Foundation titled “Rethinking the Path from Evidence to Decision-Making.” The workshop included presentations, discussions, and expert panels led by world-renowned international experts in the field of evidence-informed policymaking (Additional file 1). The workshop’s intended outcomes were threefold:

1. To Increase knowledge and skills in evidence-informed policy among the workshop's participants, including policymakers, researchers, and journalists;
2. To develop a research agenda and strengthen collaboration between experts internationally; and
3. To create a community for sharing experience, research, and best practices in Evidence-Informed Policymaking.

## The opening plenary: setting the stage

The workshop opened with plenary lectures from Ran Balicer, the Chief Innovation Officer of Clalit Health Services, John Lavis, founder and director of the McMaster Health Forum, and Tanja Kuchenmüller, Unit Head of the Evidence to Policy and Impact Unit at the World Health Organization (WHO). Balicer discussed that scientists are accustomed to taking particular care in their statements and are hesitant to make sweeping, strong statements. He emphasized that this approach is perceived as a “grey” message when communicating to the public and decision-makers and that it is necessary to communicate a clear, accurate bottom line during these communications.

John Lavis iterated that we currently have a once-in-a-generation opportunity to work with political leaders to build on the “right” lessons learned from our own and other jurisdictions. Lavis emphasized the need for context-specific rapid jurisdictional assessments to identify what works well and what gaps exist in domestic evidence-support systems. Lavis encourages using a “rapid learning and improvement” approach in the remainder of 2022 to systematize (scale up) what is working well and fill the gaps in these systems.

Tanja Kuchenmüller presented WHO’s evidence-to-policy work, in particular the Evidence-informed Policy Network (EVIPNet) [19], a global initiative using cutting-edge methods to knowledge translation for better health policy-making. ​WHO EVIPNet supports countries in mobilizing the best available evidence and establishing related systems and infrastructures to deliver high-quality, effective policies and sustainably strengthen national health systems. Worldwide more than 50 member countries are part of the EVIPNet. With its Call for Action, launched at the 2021 WHO Global Evidence-to-Policy Summit [20], EVIPNet invites governments, intergovernmental organizations, and other key stakeholders to join the network and put in place implementation plans and dedicated resources for each of these four main recommendations [17]. Furthermore, a key resource has recently been published by WHO for evidence-informed decision-making [18]. The new WHO Guide provides systematic guidance to all who (i) need to create, commission, fund, broker, or apply evidence in policy and practice and (ii) aim to establish closer collaboration across the different workstreams of the evidence ecosystem. It comprises a textbook on evidence-informed decision-making, an evidence ecosystem framework for the impact​, which serves as a matrix to capture WHO’s methods and tools linked to promoting better-informed decision-making in countries and globally. The guide also serves as a toolbox supporting the operationalization​ of evidence-informed decision-making, complemented by an online repository of evidence-informed decision-making tools [18].

These opening lectures set the tone for the next day and a half in which researchers, policymakers, students, journalists, and leaders across various disciplines met to explore and consider how evidence-informed policymaking can be improved in Israel on the macro, meso, and micro levels.

## Communicating to policy makers

A major goal of the workshop was understanding the issues involved with communication between policymakers and researchers. Workshop participants broke into group discussions to explore some of the nuances and challenges of this communication. The first group discussion was led by Tanja Kuchenmüller and Jorge Barreto, a public health researcher and EVIPNet Global (WHO) steering group member, and addressed communicating research to policymakers, based on the findings from a recent rapid scoping review regarding research communication guidance, tools, and frameworks [1]. The group opened with an exercise in which participants drew images of what came to mind when they thought of communicating with policymakers. Many participants depicted imagery connected to barriers and frustration, such as a wall between the policymakers and researchers or a researcher shouting his findings to a policymaker who does not have ears. After this discussion of introductory themes, Jorge presented the findings of his recent scoping review that investigated policymakers’ perspectives and preferences regarding research evidence communication. The main theme from the scoping review is that communication between policymakers and researchers is an integrated process. Participants emphasized that even if a scientific message is clear, policymakers’ “ears may be closed” since no foundational relationship exists. The latter and, in particular, early exchange and collaboration between policymakers and researchers are often essential for effective communication.

## The science and art of science communication

The second group session, led by Bruce Lewenstein, a professor of science communication at Cornell University, and Julia Belluz, an expert in science communication in journalism, discussed the nuances of communication between scientists, journalists, and different audiences. It was widely accepted that researchers and journalists must collaborate to effectively share scientific information with the public. However, each stakeholder has distinct roles and encounters challenges in science communication. From the researchers’ perspectives, challenges included thinking outside the disciplinary mindset, adjusting to short time frames (seizing the moment), and learning how to transform evidence into stories. For journalists, a lack of prior knowledge in the reported field, requirements to deliver sensational news, and converting messages from the scientific literature to social media platforms were major challenges. Tensions were discussed between science being developed by a professional elite and the idea that “science is too important to be left to the scientists” [3]. Also, the participants acknowledged that “tips” from scientists to journalists, and vice-versa, on how to do their job better might feel intrusive and inappropriate. Bearing these challenges and gaps in mind, the group discussed methods for training researchers and journalists in each other’s fields and frameworks for sharing knowledge and tools. Special emphasis was placed on navigating training and its timing and context. In addition, the group highlighted the importance of cultivating long-term relationships between researchers and journalists to bridge the inter-professional gap in science communication.

## Science infographics and data visualization

Science infographics and data visualizations are powerful tools for shaping public opinion and policy. The third group discussion was led by Mushon Zer-Aviv, a designer, researcher, educator, and media activist, and Nadav Davidovitch, a professor of health policy and public health at Ben-Gurion University. During the session, participants discussed how to provide context when using infographics and ethical issues in data visualizations. For example, during the COVID pandemic, data infographics were frequently used to highlight trends in cases and mortality. However, other impacts of the pandemic on society and individuals, such as school closings and mental wellbeing, were seldom visualized. Excluding these aspects created an imbalance in the issues addressed by policymakers and sometimes did not allow for policies to be presented and understood in a broader contextualization. A responsible and ethical approach to data visualization needs to foresee the impact of “viral infographics” on human behavior and design infographics to have a positive influence. Acknowledging the complexity of this mission, the participants underscored the importance of roundtable discussions among designers, health professionals, social scientists, and public representatives when forming data visualizations. Also, designing data visualizations and science infographics were appreciated as specialties that need to be learned and taught through multi-disciplinary courses, including schools of public health and schools of art.

## State of the art in evidence-informed policy: challenges and opportunities

The day continued with a panel discussion led by Moriah Ellen, including experts from the World Health Organization, the McMaster Health Forum, and Ben-Gurion University. During the panel discussion, a debate developed between members of the panel discussion regarding whether and how values play a role when conveying evidence. It is well established that knowledge alone cannot influence policy change in complex political environments. Rather, the evidence must be accompanied by several elements, including but not limited to policymaker heuristics, successfully maintained networks, and persuasive narratives [14]. However, to what extent should a person’s values play a role in the process?

John Lavis agrees that understanding the interplay between technical, social, and political values is critical for policy analysis and designing appropriate policies [15]. However, he firmly believes that the personal values of researchers and evidence intermediaries must remain irrelevant while conducting, synthesizing, and presenting evidence. Even though values play a big role in making policy decisions, Lavis insists that we must demand more from the experts we see on TV. “I really want them to be able to tell me what they are saying based on the best evidence… we should have a standard where we ask them to tell us how they identified, assessed, and interpreted the evidence that led them to what they are saying.”

Conversely, while Nadav Davidovitch agrees that experts need to base their statements on the best available evidence, he maintains that “evidence and what kind of data you want to collect is already based on your values.” For example, the researcher’s answer to the question, “Is health a right or a commodity?” affects how he or she designs the study, collects the data, and interprets the information. Davidovitch encourages experts to be upfront and transparent about their values when presenting the data, as he sees the two as intrinsically connected.

## Proposed recipe for impact

Using compelling case studies on topics ranging from the global anti-vaccine movement to America's staggering maternal mortality problem, Julia Belluz went beyond the daily headlines to take a deeper look at science communication from the journalist's perspective. Corresponding to the group discussion held during the workshop, Julia proposed the following "Recipe for Impact":

- 3 cups of evidence
- 3 cups of well-chosen stories
- One tablespoon of political will
- A sprinkle of good timing and a dash of luck.
- Bake into the public conversation.

Her point was that evidence alone is insufficient to move people and change policy. It would be best if you also had illustrative stories and, for real impact, political will about the issue you are reporting on, along with some luck and good timing. While ingredients and ratios can be debated, Julia’s proposed recipe illustrates the need to combine facts with narratives and other elements when communicating scientific evidence to a wide audience. In her presentation, Julia also emphasized the need for collaboration among researchers—who produce the evidence—and journalists—who humanize the information with stories.

## The next generation for intervention and research in evidence-informed policy

Day two of the workshop continued with a roundtable exercise dedicated to exploring several domains and processes necessary for institutionalizing evidence-informed policymaking. Tanja Kuchenmüller, Marge Reinap, and Mark Leys led the group discussions. The session was founded in the WHO EIP institutionalization framework that comprises six domains: (1) governance; (2) standards and routinized processes; (3) leadership and commitment; (4) resources and capacity building/strengthening; (5) partnership, collective action and support; and (6) culture [7, 8]. Table 1 includes summarized points on the WHO EIP institutionalization framework roundtable discussions. In addition, two of the group discussions are presented below in greater depth.

**Table 1:** Group summaries of the WHO EIP institutionalization framework roundtable

Group title and leader	Takeaway Messages:	Implications for Israel
Institutionalization	There is a need to assess and create agile, multisectoral EIP structures and processes that provide contextualized evidence on high policy priorities Lessons for multisectoral work can be learned from the health sector	Israel must strengthen partnerships and collaborations between Health Ministry, HMOs & international organizations in EIP Chief scientists should be regrouped from different sectors to create a joint EIDM system and showcase leadership and commitment in this field at the national level
Norms, standards, and tools	To succeed at evidence implementation, there is a need for transparency, accountability, collaboration Health must be valued by society beyond finance	Israel must adapt standards for evaluation for program implementation Global standards should be adapted to local Israeli law Local influencers and opinion leaders should be utilized as a tool for evidence promotion
Capacity	Evidence capacity must be built among potential intermediary influencers such as lobbyists, NGOs, reporters, researchers, policymakers To build capacity, citizen and stakeholder engagement must be integrated	Israel needs more EIP training (e.g., evidence workshops and seminars) Israeli journalists and communication scientists should be trained in EIP principles and methods
Timely, relevant, accessible evidence	To develop timely, relevant, accessible evidence, different types of data must be utilized, including international and local Data must be provided, organized, and synthesized before being presented to relevant parties Evidence integration is a technical, methodological, and content issue	Israel must map the current situation to understand which tools and systems for EIP exist and which do not Israel must focus on a problem-oriented approach and try to establish relevant evidence

*EIDM* Evidence-Informed Decision Making, *EIP* Evidence-Informed Policy, *HMO* Health Management Organizations, *WHO* World Health Organization, *NGO* Non-Governmental Organizations

## Group discussion on institutionalization, governance, and partnerships

As defined by the WHO EIP institutionalization framework, governance relates to the rule-making and steering-related functions that promote the institutionalization of evidence-informed policymaking, such as knowledge translation platforms that connect research and policy [7, 8]. The creation of these platforms enhances the likelihood that knowledge transfer systems are protected despite political changes [10].

This group discussion emphasized the importance of creating and improving multi-sectoral partnerships, such as engaging the Ministry of Finance in policy decisions beyond economics. Similarly, partnerships between the Ministry of Health and the HMOs are essential due to the HMOs’ data and policy influence.

A suggestion was put forth to coordinate between the chief scientists of different sectors to create a joint evidence-informed policymaking system to showcase EIPM leadership and emphasize a commitment to promote the routine use of evidence at the national level. Group members suggested that policymakers take lessons from the multisectoral work utilized in the health sector and implement similar practices in policy areas beyond health.

Group members also highlighted the need to create an interagency platform that can serve as a safe space for actors to convene and deliberate on policy issues. The EVIPNNet situation analysis was posited as a potentially helpful tool to identify relevant stakeholders for such a platform [21]. The group also concluded that any future EIP advisory body must be hosted at the prime minister level.

## Group discussion on building capacity in evidence-informed policy

The group discussed several questions regarding capacity building in evidence-informed policy: (1) Building capacity of whom? (2) What capacities need to be built? (3) How to provide capacity building? And (4) how to ensure effectiveness, impact, and sustainability.

Regarding the first question, key stakeholder groups include researchers, policymakers, and intermediary influencers. The latter group encompasses journalists, lobbyists, NGOs, and state auditors. Specific emphasis was placed on capacity building for young professionals, such as student researchers, internship participants, and middle managers.

Capacities that need to be built include storytelling and sloganizing, knowledge translation, and stakeholder engagement. Specifically, how to engage with citizens and decision-makers using science infographics and data visualizations should be prioritized as part of capacity building. Also, the group discussed the importance of rethinking the roles of chief scientists and better equipping them with relevant tools, so they may effectively serve as intermediaries between scientists and decision-makers.

Capacity building should encompass evidence-informed policy (EIP) training by dedicated centers, academic education (i.e., in science communication departments), training of trainers programs, and peer-to-peer role modeling. Training of trainers programs was deemed especially relevant for middle managers in government offices. Also, e-training tools in EIP should be developed and incorporated into capacity-building initiatives. The group also discussed the need to tailor scaled capacity building according to individuals’ levels and needs, including those who develop their careers in the field of EIP.

## Lessons learned: implications for evidence-informed policy in Israel

The lessons, insights, and conclusions from this two-day workshop were numerous and far-reaching. They can be summarized by how they pertain to the macro (institutionalization), meso (capacity), and micro (relational) levels.

On the **macro** or institutionalization level, the workshop identified the dire need to create stable systems and a sustainable environment for evidence-informed policy in Israel. The evidence systems created in Brazil serve as an excellent example of EIP institutionalization that remains in place, separate from changing political realities. The Brazilian evidence centres address varied health initiatives, from the COVID-19 rapid evidence summary series to health issues among indigenous communities [16]. A similar example of EIP institutionalization on the macro level and its positive impact is the role of Anthony Fauci as head of the NIAID. Despite the extreme partisanship associated with coronavirus policy in the US, American citizens across both sides of the political aisle trusted medical advice from Dr. Fauci [2].

In the Israeli context, the Israel Institute of Health Policy Research’s stable budget, which is required by the National Health Law (1995), is a positive example of the type of institutionalization necessary to sustain EIP best practices. The law (Section 52b) requires that the Health Council carry out research, surveys, and professional expert opinions on health policy research and promises sustained funding to ensure this occurs [11]. This law helps ensure that institutions promoting evidence-informed policymaking stay in place despite the shifting political environment. However, there remain several missing functions regarding EIP institutionalization in the Israeli system, particularly in the lack of evidence synthesis on the national and macro levels. Israel must create sustainable systems that will remain in place and create a lasting impact for EIP. The country needs to create systems that assess the current decision-making processes. Also, there is a need for agile, multisectoral EIP structures and processes that provide contextualized evidence on high policy priorities in Israel.

On the **meso** or capacity level, participants identified the dire need for academic programs to train people proficient in multiple disciplines related to evidence-informed policy and science communication, such as public health, communication, social marketing, infographics, and arts. Some notable examples of this in Israel include Ayelet Baram Tazabari’s research group in the Technion and publications by the Davidson Institute at the Weizmann Institute of Science. However, despite these examples, a significant gap exists between the aforementioned fields, and the Israeli academic landscape would benefit by integrating novel programs and courses to combine these disciplines.

Lastly, on the **micro** level, the EIP workshop helped highlight the pressing need to foster professional relationships and training between scientists and journalists in Israel. Throughout the workshop, the sentiment arose that the current state of the relationships between Israeli scientists and journalists is sub-optimal, and efforts must be made to foster and strengthen these relationships. Going forward, policymakers, researchers, experts, journalists, and public members must work together to cultivate each others’ skills while creating an environment of collaboration and partnership in which each player’s expertise is respected. Only through shared goals of creating, synthesizing, implementing, and communicating the best possible evidence to the public can we hope to foster an environment that prioritizes decisions based on evidence and change Israeli society for the better.

Looking forward, IS-PEC plans to collaborate with government agencies and lead projects in the evidence-informed policy field, both in Israel and in collaboration with international partners. This includes the formation of a steering committee dedicated to improving evidence-support in government ministries, a rapid jurisdictional assessment of existing evidence-support systems throughout the Israeli government and with external partners, and conducting symposia on knowledge transfer and exchange for chief scientists and their staffs. IS-PEC benefits from the expertise of international scholars and practitioners of evidence-informed policy and the support of parallel centers worldwide. Due to its interdisciplinary expertise, the long and diverse experiences of its members, being trusted and well-situated within the Israeli context, and commitment to improving the uptake of evidence-informed policy-making, IS-PEC is uniquely positioned to advance the field in Israel and abroad with the international EIP community.

## Supplementary Information

**Additional file 1.** The workshop included presentations, discussions, and expert panels led by world-renowned international experts in the field of evidence-informed policymaking.

## Data Availability

The workshop material is available at https://in.bgu.ac.il/en/is-pec/Pages/default.aspx
