# Supplementary material for: Rethinking the path from evidence to decision-making
Source: Isr J Health Policy Res. 2023 Mar 27;12:10. doi: 10.1186/s13584-023-00559-8 (PMC10041505; doi:10.1186/s13584-023-00559-8)
Supplement: Supplementary file 1 — Additional file 1. The workshop included presentations, discussions, and expert panels led by world-renowned international experts in the field of evidence-informed policymaking. [file 13584_2023_559_MOESM1_ESM.docx]

**Appendix 1:** Short introductions of the conference speakers, in alphabetical order

*
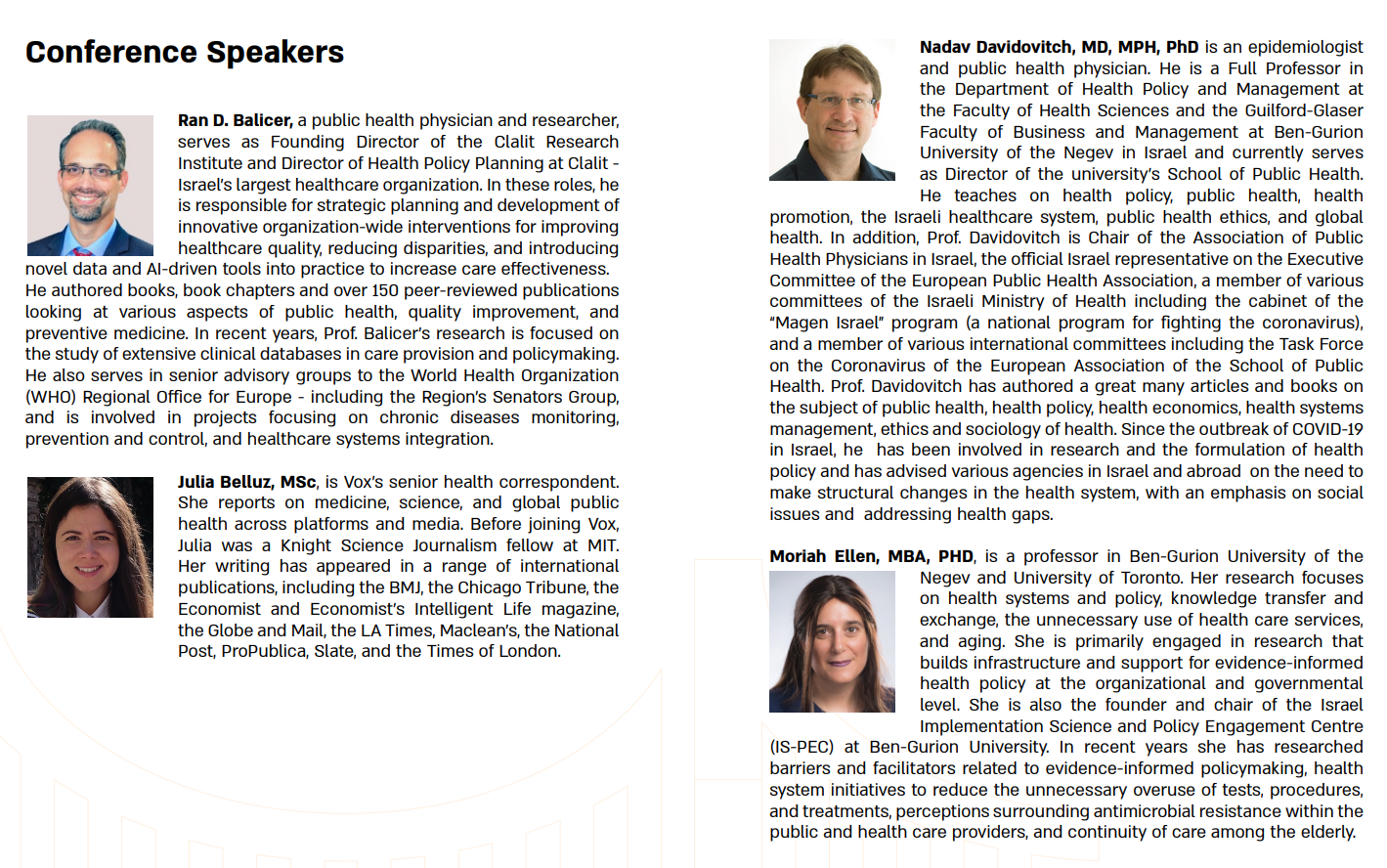
*

*
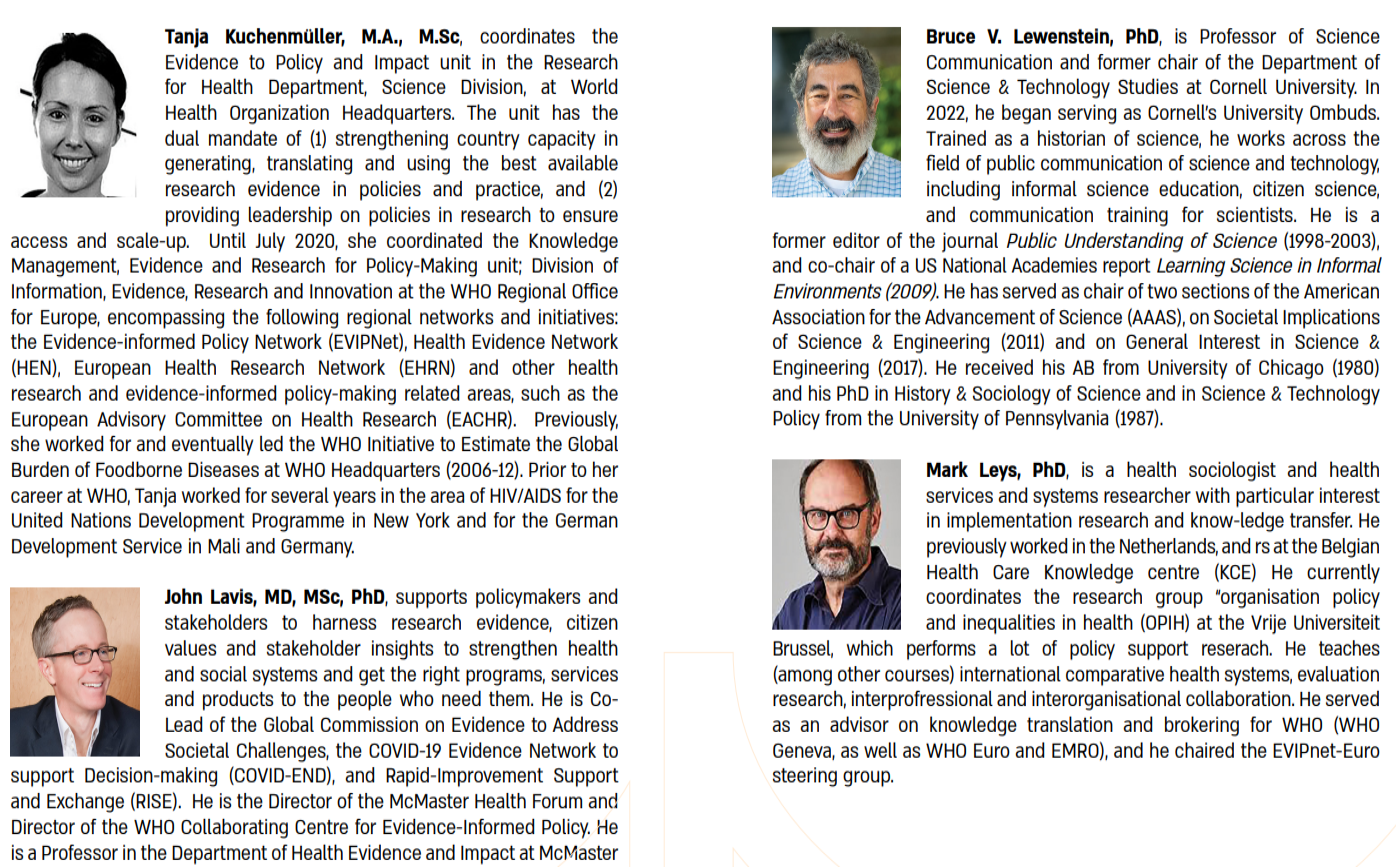
*

**Conference speakers – continued***
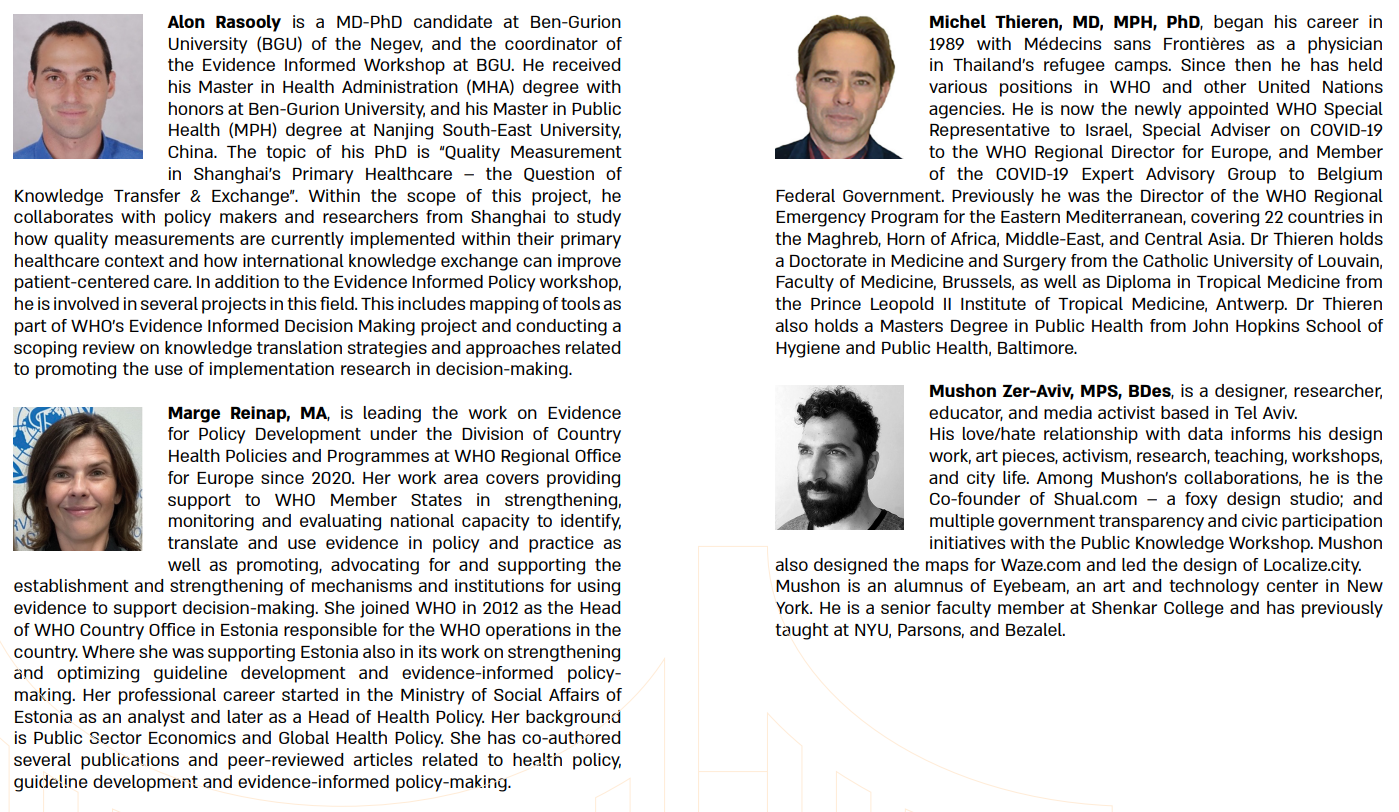
*
